# 511. Risk Factors for Seeking Medical Care Following Nirmatrelvir-Ritonavir (Paxlovid) Treatment For COVID-19

**DOI:** 10.1093/ofid/ofad500.580

**Published:** 2023-11-27

**Authors:** Ashish Bhargava, Susan M Szpunar, Mamta Sharma, Louis Saravolatz

**Affiliations:** Ascension St. John Hospital; Wayne State University School of Medicine, Grosse Pointe Woods, Michigan; Ascension St. John Hospital, MI; Ascension St John Hospital & Medical Center, Grosse Pointe Woods, MI. 48236, Michigan; Ascension St John Hospital, gross pointe woods, Michigan

## Abstract

**Background:**

In December 2021, the FDA issued an emergency use authorization (EUA) for nirmatrelvir-ritonavir (Paxlovid). This was based on the randomized placebo-controlled trial data from the EPIC-HR study which showed that high-risk non-hospitalized patients given Paxlovid had an 88.9% reduction in the relative risk of progression to hospitalization or death by Day 28 in coronavirus disease 2019 (COVID-19). We aimed to study the risk factors for seeking medical care following Paxlovid treatment.

**Methods:**

In this historical cohort study, we assessed risk factors for high-risk non-hospitalized patients treated with Paxlovid from January 1, 2022, to December 31, 2022, at outpatient facilities associated with Ascension St. John Hospital. Our outcome variable was the composite of subsequent evaluation in the Emergency Department (ED) or inpatient admission within four weeks of their Paxlovid treatment. Data were analyzed using SPSS v. 29.0. The study was approved by the IRB.

**Results:**

Of 371 patients who received Paxlovid treatment, the mean (SD) age was 59.4 (±13.8) years; 63.9 % (237) were female, and 77.7% (282) white. The mean body mass index (BMI) of the cohort was 31.3 ± 7.8 kg/m2. The incidence of the composite event was 6.7% (25/371). The risk of the composite event was higher in patients with a history of myocardial infarction (MI) (28 % vs 9.3%), congestive heart failure (CHF) (12 % vs 2.3%), chronic lung diseases (CLD) (48 % vs 26.4%) and diabetes mellitus (DM) with complication (24% vs 4.1%), respectively. Alcohol users were less likely to seek medical care following Paxlovid (40 % vs 63.7%, respectively). There were no differences in BMI, tobacco use or vaccination rates between the groups. In multivariable logistic regression, factors associated with seeking medical care post-Paxlovid were female sex (OR 4.6; 95% CI 1.4-15.3; p= 0.013), alcohol use (OR 0.4; 95% CI 0.2-0.9; p= 0.039), MI (OR 4.0; 95% CI 1.4-11.8; p=0.011), DM with complications (OR 6.9; 95% CI 2.0-23.3; p= 0.002), CLD (OR=3.9, 95% CI 1.1-13.5; p=0.03), at the time of Paxlovid treatments.

**Conclusion:**

Independent risk factors for seeking medical care following Paxlovid treatment for COVID-19 were MI, CHF, CLD, and DM with complications while alcohol users were less likely to seek medical care following the Paxlovid treatment.

**Disclosures:**

**All Authors**: No reported disclosures

